# Comment on “Intraoperative Contrast Enhanced Ultrasound Evaluates the Grade of Glioma”

**DOI:** 10.1155/2017/3570895

**Published:** 2017-06-04

**Authors:** Raman Mahalangikar, Sumit Sinha, Rajeev Sharma

**Affiliations:** Department of Neurosurgery, Neurosciences Centre, All India Institute of Medical Sciences, Ansari Nagar East, New Delhi 110029, India

We read the article “Intraoperative Contrast Enhanced Ultrasound Evaluates the Grade of Glioma” by Cheng et al. published in March 2016 [1]. The aim of this study was to evaluate the use of intraoperative contrast enhanced ultrasonography (CEUS) to assess the grade of glioma and correlation of microvascular density (MVD) and vascular endothelial growth factor (VEGF) expression with CEUS for glioma.

In this study, 88 patients diagnosed with supratentorial glioma with CT/MRI underwent intraoperative CEUS. The authors have mentioned in their article that patients aged from 18 to 69 years were included with mean age of 45 ± 12.8 years in the Materials and Methods, whereas, in the Results, they have mentioned patients aged from 20 to 69 years with mean age of 47.9 ± 11.4 years. The presented data regarding demographic characteristics of patients included in this study appears to be conflicting.

Patients with a solitary tumour of diameter from 2.1 to 5.4 cm were included in this study. The authors did not mention the reason for inclusion of patients with tumours within this given range of diameter.

Ultrasound is a highly operator dependent modality and the interpretation is highly variable depending upon the experience and the technical training of the personnel involved. In this study, although the intraoperative conventional ultrasonography (CUS) and CEUS were performed by a single doctor, the authors did not mention the experience and his technical level of expertise in performing the diagnostic procedure. In this study different characteristics of tumour were noted by a single observer. This observation can be a source of examiner bias. Rather, two independent observers performing the study with evaluation of interobserver agreement on the grade of glioma would be an appropriate strategy and would have made the study design robust. Data regarding characteristics of tumours should have been tabulated and calculation of any significant difference between characteristics of low grade glioma and high grade glioma would have given a more appropriate result.

Selection of region of interest (ROI) during image analysis has no fixed criteria in this study. The ROI was a circular area of 0.9 cm diameter without any necrosis. Selection of ROI at the centre of tumour and at the periphery of tumour can affect the measurements on CEUS, which were the time to start (TTS), the time to peak (TTP), and the absolute peak intensity (API) because of heterogeneity of vascularity in the tumour [2].

Unfortunately for the authors, there appears to be misprinting in positive grading criteria for expression of VEGF. Positive cell rate of 11 to 40% was printed as (+) and 41 to 75% was also printed as (+) in this article.

All CEUS procedures were carried out in patients who underwent general anesthesia for surgery. The authors have mentioned there were no adverse reactions for contrast during and after examination (such as dizziness, headache, abdominal pain, feeling strange, joint and muscle pain, and weakness). The adverse effects that authors mentioned cannot be assessed in patients under general anesthesia. The criteria set by authors to assess adverse reaction for contrast during the procedure are not mentioned in this article.

The authors have calculated MVD and VEGF grading for 18 patients of LGG and 24 patients of HGG whereas the study had 50 cases of HGG and 38 cases of LGG. It can be possible that the authors have randomly selected the patients for this outcome. But then the presentation of data on MVD and VEGF in selected patients can be a source of bias which can significantly affect the results. The conclusion should have only been drawn if MVD and VEGF expression was measured in all cases. This also puts the conclusion of this study in question, whether there was really a positive correlation between API and MVD and VEGF.

The authors aimed to assess the grade of glioma with the use of intraoperative CEUS. This study does not give any information on sensitivity, specificity, positive or negative predictive value of intraoperative CEUS on grade of glioma.

## Abbreviations

API: The absolute peak intensity
CEUS: Contrast enhanced ultrasonography
CT: Computed tomography
CUS: Conventional ultrasonography
HGG: High grade glioma
LGG: Low grade glioma
MRI: Magnetic resonance imaging
MVD: Microvascular density
ROI: Region of interest
TTP: The time to peak
TTS: Time to start
VEGF: Vascular endothelial growth factor.
